# From Harassment to Harmony: The Mediating Role of Organizational Tolerance and Moderation of Quality of Supervisor–Subordinate Guanxi Among Chinese Law Enforcement Workers

**DOI:** 10.1002/pchj.70009

**Published:** 2025-04-03

**Authors:** Tao Liang

**Affiliations:** ^1^ East China University of Political Science and Law Shanghai China

**Keywords:** Chinese law enforcement officers, organizational tolerance, quality of supervisor‐subordinate guanxi, turnover intention, workplace harassment

## Abstract

Workplace harassment has garnered considerable attention in organizational research due to its profound impact on employee well‐being and organizational outcomes. Harassment in the workplace can take various forms, including verbal abuse, bullying, and other forms of psychological aggression, which collectively contribute to a toxic work environment. Such negative experiences can lead to severe consequences, including diminished job satisfaction, increased stress levels, and a heightened intention to leave the organization. The present study aims to test the impact of workplace harassment on employees' turnover intentions, highlighting the mediating role of organizational tolerance and the moderating effect of supervisor–subordinate guanxi. Additionally, the study considers the influence of gender and professional category to be covariates in the analysis. The sample for this study comprises 821 law enforcement officers (58.5% male) from various agencies within China. Workplace harassment was found to significantly and positively correlate with employees' turnover intentions. Organizational tolerance mediated this relationship. While the direct moderating effect of supervisor–subordinate guanxi was not statistically significant, the quality of guanxi moderated the mediated pathway. High‐quality supervisor–subordinate guanxi reduced the negative impact of workplace harassment by weakening the mediating role of organizational tolerance. Gender and professional category significantly influenced the results, with women and lower‐ranking officers demonstrating higher sensitivity to workplace harassment and organizational tolerance, thereby exacerbating turnover intentions. The findings underscore the importance of addressing workplace harassment and fostering high‐quality supervisor–subordinate relationships to mitigate its adverse effects. Furthermore, targeted interventions should account for the unique vulnerabilities associated with gender and professional category to enhance retention strategies and foster supportive organizational environments.

## 1 Introduction

Workplace harassment has garnered considerable attention in organizational research due to its profound impact on employee well‐being and organizational outcomes. Harassment in the workplace can take various forms, including verbal abuse, bullying, and psychological aggression, which collectively contribute to a toxic work environment. Such negative experiences can lead to severe consequences, including diminished job satisfaction, increased stress levels, and heightened turnover intention (Einarsen et al. 2020). This study seeks to explore the intricate dynamics between workplace harassment and turnover intention among law enforcement officers in China, a sector where the hierarchical and high‐stress nature of the job may exacerbate these issues (Zhong et al. 2023).

In the context of law enforcement, the effects of workplace harassment can be particularly pronounced due to the demanding and often hazardous nature of the work (Wu et al. 2020). Officers operate under significant stressors, both internal and external to their organizations, while being expected to maintain high levels of performance and professionalism. Understanding the factors that contribute to turnover intention in this field is crucial for developing effective retention strategies and ensuring organizational stability (Wang et al. 2021). Demographic factors, such as gender and professional category, also play critical roles in shaping employees' perceptions of workplace harassment. Research has shown that women are more likely to experience harassment in male‐dominated environments, leading to amplified turnover intentions (Glomb et al. 1997; Lim and Cortina 2005; Zhou, Nguyen, et al. 2024). Similarly, employees in lower professional ranks often report a greater prevalence of harassment due to power imbalances within hierarchical structures (Ajuwa et al. 2024).

Organizational tolerance refers to the extent to which an organization condones or fails to address incidents of harassment. When harassment is perceived to be tolerated, it can exacerbate turnover intentions by signaling a lack of organizational support and protection (Martinez 2023). This study hypothesizes that higher levels of perceived organizational tolerance for harassment will strengthen the positive relationship between workplace harassment and turnover intention, advancing theoretical understanding of how organizational culture shapes employee behavior.

Furthermore, the quality of supervisor–subordinate guanxi, a concept rooted in Chinese cultural practices, is investigated as a moderating factor. Guanxi refers to the interpersonal relationships and social networks that influence business and social interactions in China. High‐quality guanxi between supervisors and subordinates fosters a supportive and trusting environment, potentially mitigating the negative impacts of workplace harassment (Yang et al. 2024). This study explores whether strong supervisor–subordinate guanxi can buffer the adverse effects of workplace harassment on turnover intention, both directly and indirectly through organizational tolerance. By integrating the cultural construct of guanxi, this research contributes to the theoretical understanding of how cultural and relational dynamics interact with workplace harassment.

By focusing on law enforcement officers in China, this study provides valuable insights into how cultural, organizational, and demographic factors interact to influence employees' responses to workplace harassment. The findings are expected to enrich the broader literature on workplace harassment and employee turnover by offering theoretical contributions regarding the roles of organizational tolerance and guanxi. Additionally, this research has practical implications for organizational policies and management practices aimed at reducing turnover rates and improving the work environment in law enforcement agencies.

In summary, this study addresses the following research questions:

- Does workplace harassment directly increase turnover intention among law enforcement officers?
- Does organizational tolerance mediate the relationship between workplace harassment and turnover intention?
- Does the quality of supervisor–subordinate guanxi moderate the direct relationship between workplace harassment and turnover intention?
- Does the quality of supervisor–subordinate guanxi moderate the indirect relationship between workplace harassment and turnover intention through organizational tolerance?

By answering these questions, this study provides a comprehensive understanding of the factors influencing turnover intentions in a high‐stress, hierarchical sector, offering guidance for effective organizational interventions and enriching the theoretical frameworks that underpin these dynamics.

### 1.1 Workplace Harassment Directly and Positively Impacts Employees' Turnover Intention

Workplace harassment encompasses a range of unwelcome behaviors that create an intimidating, hostile, or offensive work environment (Xie and Zheng 2023). This can be based on race, sex, religion, national origin, age, disability, sexual orientation, or other personal attributes. Harassment becomes a significant legal concern when enduring such behavior becomes a requisite for continued employment or when the behavior is severe or pervasive enough to be considered by a reasonable person as creating a hostile work environment. Sexual harassment, a commonly recognized form, includes unwelcome sexual advances or remarks (Liang 2024). Additionally, harassment can also occur in digital formats, where inappropriate or threatening communications are sent through electronic means, impacting the victim even beyond the physical workplace. Effective organizational management against harassment involves the implementation of strict policies, regular training, and a clear process for handling complaints, which together foster a safe and respectful working environment.

Workplace harassment has a significant and direct impact on turnover intention among different kinds of employees. Several studies highlight this relationship, emphasizing the detrimental effects of harassment on employee retention and organizational commitment (Zhong et al. 2023).

Workplace harassment constitutes a critical concern, potentially influencing employee turnover intentions (Nguyen et al. 2023). In the context of Chinese law enforcement, this issue acquires specific cultural and organizational dimensions that merit close examination. Workplace harassment in law enforcement not only undermines professional conduct but also affects the personal and emotional well‐being of officers. Studies have shown that police work, by its very nature, exposes officers to high‐stress situations which, when coupled with internal factors such as workplace harassment, can lead to significant psychological stress (Zhou, Li, et al. 2024). Such environments are potent catalysts for increased turnover intentions, as they compromise an employee's ability to perform effectively.

The Chinese cultural context offers a unique perspective on authority and hierarchy, influencing how harassment is perceived and addressed (Gui 2023). In traditional Chinese organizations, hierarchical relationships are strongly emphasized, which may prevent victims of harassment from speaking out due to fear of reprisal or loss of face (Wong et al. 2002). This dynamic is particularly pronounced in law enforcement agencies where the chain of command is rigid, thus potentially exacerbating the negative impacts of harassment on employee morale and intention to remain in the position. Despite the growing recognition of workplace harassment as a critical issue in organizational research, limited attention has been paid to how these cultural and hierarchical factors shape the experience and consequences of harassment in Chinese law enforcement.

Existing empirical research has demonstrated a direct correlation between workplace harassment and turnover intentions across various occupational fields in China (Ma et al. 2024; Ren and Kim 2023; Xia et al. 2023). Specific studies involving Chinese police officers have highlighted that experiences of workplace harassment not only increase the likelihood of developing strong turnover intentions but also undermine critical aspects of their professional identity and organizational attachment. For instance, Ha et al. (2024) found that cumulative stress from harassment leads to emotional exhaustion, reducing officers' willingness to engage proactively with their roles. Furthermore, such stress disrupts trust in supervisory relationships and weakens group cohesion, which is essential for effective law enforcement operations. These findings underscore the importance of addressing workplace culture and interpersonal dynamics to mitigate the adverse effects of harassment.

However, research gaps remain in understanding the mechanisms through which workplace harassment influences turnover intentions, particularly in high‐stress, hierarchical environments such as law enforcement. This study addresses these gaps by investigating the mediating role of organizational tolerance, defined as the degree to which harassment is perceived to be condoned or inadequately addressed within an organization. While existing studies highlight the detrimental effects of perceived organizational tolerance (Martinez 2023), limited research has explored its specific impact within law enforcement agencies or its interaction with cultural and structural factors.

Another novel contribution of this study lies in examining the moderating role of supervisor–subordinate guanxi, a construct rooted in Chinese culture that reflects the quality of interpersonal relationships. While guanxi has been extensively studied in business contexts, its potential to buffer the negative effects of harassment within law enforcement has received scant attention. This study aims to fill this gap by exploring whether high‐quality guanxi can mitigate the adverse impacts of harassment, both directly and indirectly through organizational tolerance.

In conclusion, workplace harassment in Chinese law enforcement significantly impacts turnover intentions, as evidenced by prior research. However, this study advances the literature by examining the mechanisms and cultural factors that exacerbate or buffer these effects. By focusing on organizational tolerance and *guanxi* as mediating and moderating variables, respectively, the study provides a deeper understanding of the interplay between workplace harassment, organizational culture, and employee outcomes in a culturally unique and high‐stress sector. These insights highlight the need for targeted interventions that account for structural, cultural, and relational dynamics in addressing workplace harassment and its consequences.

### 1.2 Organizational Tolerance Mediates the Relationship Between Workplace Harassment and Employees' Turnover Intention

Organizational tolerance refers to the extent to which inappropriate behaviors, including harassment, are overlooked, ignored, or tacitly approved within the workplace (Bostelman 2020). A high level of organizational tolerance for harassment often signals to employees that such conduct is acceptable or at least not sufficiently problematic to warrant correction or censure. This perception can profoundly impact employees' morale and their ongoing commitment to the organization (Ford and Ivancic 2020).

When an organization exhibits a high tolerance for harassment, it may inadvertently encourage a culture where negative behaviors flourish, leading to increased emotional and psychological strain on employees. Research indicates that the perception of organizational tolerance of harassment correlates strongly with heightened turnover intentions among employees. Employees are more likely to consider leaving an organization if they feel unprotected and unsupported in their workplace (Schat 2005). This response is particularly pronounced when employees see little to no effort from management to address or mitigate harassing behaviors.

Management may “turn a blind eye” to harassment for various reasons, often tied to organizational and cultural dynamics (Einarsen et al. 2020). For instance, leadership might prioritize short‐term productivity or team performance over addressing problematic behaviors, particularly in high‐stress and hierarchical environments like law enforcement (Hershcovis et al. 2021). In such contexts, addressing harassment may be perceived as disruptive or even threatening to organizational cohesion. Additionally, managers may lack the necessary training, resources, or institutional support to confront such issues effectively (Beaton et al. 2021). Furthermore, a workplace culture that normalizes harassment or views it as a byproduct of challenging work conditions can exacerbate the problem, allowing such behaviors to persist unchallenged (Bergenfeld et al. 2021). These organizational dynamics not only perpetuate harassment but also erode trust, morale, and overall employee well‐being, highlighting the critical need for proactive management and robust anti‐harassment policies (Lu and Luqiu 2023).

Several studies have quantitatively assessed the mediating role of organizational tolerance in the harassment–turnover intention nexus. For instance, recent studies demonstrate that organizational tolerance not only amplifies the direct effects of harassment on turnover intentions but also serves as a critical mediating variable that explains much of the variance in turnover intention rates among employees subjected to harassment (Kaur 2023; Megeirhi et al. 2020). This evidence underscores the necessity for organizations to adopt zero‐tolerance policies toward harassment to retain talent and maintain a healthy work environment.

To sum up, organizational tolerance significantly mediates the relationship between workplace harassment and employees' turnover intentions. Organizations that fail to address harassment adequately may see higher turnover rates, which emphasizes the importance of proactive management and robust policy frameworks to cultivate a supportive and harassment‐free workplace. This mediation effect is critical for understanding the broader implications of workplace harassment and offers a compelling argument for stringent organizational interventions.

### 1.3 Quality of Supervisor–Subordinate Guanxi Moderates the Direct Relationship Between Workplace Harassment and Employees' Turnover Intention

According to Chen and Chen (2004), the quality of guanxi is a subjective assessment of the state of personal relationships, akin to the notion of “tie strength” in network literature. High‐quality guanxi is characterized by mutual trust, reciprocal benefits, and emotional support. In the organizational context, this translates into relationships where both parties value and nurture a connection that goes beyond mere transactional interactions. The concept of guanxi, integral to Chinese social interaction and organizational behavior, encompasses the personal connections that influence social and business interactions. The quality of guanxi between supervisors and subordinates can significantly moderate the impact of workplace harassment on employees' turnover intentions (Ameyaw et al. 2022).

Following Fiske's (Fiske 1992) theory, a shift from contract‐based relationships (market pricing relationships) to communal sharing relationships within the supervisor–subordinate dynamic indicates high‐quality guanxi. In such relationships, the roles extend beyond formal job descriptions and duties to include care for each other's welfare and mutual respect, forming a strong interpersonal bond that transcends basic professional requirements (Chen et al. 2009).

The quality of the supervisor–subordinate guanxi plays a crucial moderating role in the presence of workplace harassment. When subordinates share a high‐quality guanxi with their supervisors, they are more likely to feel supported and valued despite the challenges posed by workplace harassment. This sense of support can mitigate the negative feelings associated with harassment and reduce the push factors leading to turnover intentions. For instance, a subordinate might tolerate adverse situations longer and remain committed to the organization if they experience a strong, supportive bond with their supervisor.

Research suggests that employees with strong guanxi with their supervisors report higher job satisfaction and lower turnover intentions, even when facing significant workplace stressors such as harassment (Guan and Frenkel 2019). This buffering effect underscores the importance of nurturing positive supervisor–subordinate relationships in organizational settings. Organizations should encourage supervisors to develop strong, supportive relationships with their subordinates, which can serve as a protective factor against the potential turnover intentions triggered by negative workplace experiences.

As a consequence, the quality of supervisor–subordinate guanxi significantly moderates the relationship between workplace harassment and turnover intentions (Zhang et al. 2023). A strong, positive guanxi can act as a buffer, reducing the negative impacts of harassment on an employee's decision to leave the organization. This highlights the need for organizations to cultivate environments where such relationships are prioritized and valued as part of the overall strategy to maintain employee satisfaction and retention.

### 1.4 Quality of Supervisor–Subordinate Guanxi Moderates the Indirect Relationship Between Workplace Harassment and Employees' Turnover Intention Mediated by Organizational Tolerance

In Chinese organizational settings, guanxi is a culturally rooted concept that encompasses the web of personal and professional connections influencing social and business interactions. Unlike the general concept of relationships, which may be categorized as personal‐based, work‐based, or social‐based, guanxi integrates all these dimensions and is deeply embedded in the normative framework of Chinese culture. Guanxi is governed by principles such as reciprocity, trust, and long‐term obligation, which shape how individuals navigate hierarchical and interpersonal dynamics (Yang et al. 2024).

The quality of supervisor–subordinate guanxi plays a pivotal role in moderating the indirect effects of workplace harassment on turnover intentions through organizational tolerance. While relationships in Western organizational contexts often focus on professional boundaries, guanxi extends beyond the workplace to incorporate personal and social connections, creating a unique support system for employees. High‐quality guanxi is characterized by mutual trust, respect, and an exchange of favors, offering a culturally specific framework for understanding workplace dynamics. Organizational tolerance can signal to employees that harassing behaviors are not significant enough to warrant intervention, potentially increasing turnover intentions among victims. However, the indirect impact of workplace harassment on turnover intentions through organizational tolerance is moderated by the quality of guanxi between supervisors and subordinates (Li and Zheng 2024). Strong guanxi provides emotional and psychological resources, enabling employees to feel secure and valued despite broader organizational tolerance for harassment (Yung Chou et al. 2014).

Supervisors with strong guanxi ties to their subordinates may intervene more actively in addressing harassment, altering the perception of organizational tolerance. Moreover, these relationships can foster informal support systems that compensate for the lack of formal organizational mechanisms to address harassment (Zhang et al. 2016). Previous research has shown that employees with strong supervisor–subordinate guanxi are less likely to perceive their work environment as tolerating harassment, even when organizational policies are insufficiently robust (Zhang et al. 2014).

Thus, guanxi moderates the pathway from workplace harassment to turnover intentions in several ways (Ju et al. 2023). First, strong guanxi can mitigate the psychological impact of harassment by providing subordinates with direct support and advocacy. Second, it may influence the extent to which employees interpret their organization as tolerant of harassment. Last, guanxi reinforces interpersonal trust and creates a sense of obligation, which can help employees feel more connected to their workplace despite negative experiences.

This study emphasizes that guanxi is not simply a cultural equivalent of relationships but a distinct construct with unique features specific to Chinese contexts. By focusing on guanxi as a moderator, the research highlights how cultural norms influence the interplay between workplace harassment, organizational tolerance, and turnover intentions. Understanding the role of guanxi provides valuable insights for developing culturally informed strategies to reduce turnover rates and improve workplace cohesion in Chinese law enforcement.

Based on the above literature review, the present study aims to test the following hypotheses, grounded in theoretical and empirical research:

Hypothesis 1 *Workplace harassment directly and positively impacts employees' turnover intention*.

This hypothesis is based on prior research showing that workplace harassment contributes to emotional exhaustion and diminished organizational attachment, ultimately increasing employees' intention to leave (Einarsen et al. 2020; Ha et al. 2024). Given the high‐stress nature of law enforcement, it is expected that the experience of harassment will directly lead to stronger turnover intentions among officers.

Hypothesis 2 *Organizational tolerance mediates the relationship between workplace harassment and employees' turnover intention*.

Organizational tolerance, or the degree to which harassment is perceived to be condoned, has been shown to exacerbate the effects of harassment on employees' well‐being and turnover intention (Martinez 2023). This study suggests that higher perceived organizational tolerance may strengthen the indirect relationship between workplace harassment and turnover intention by signaling a lack of organizational support and accountability.

Hypothesis 3 *Quality of supervisor–subordinate guanxi moderates the direct relationshipbetween workplace harassment and employees' turnover intention.*

Supervisor–subordinate guanxi, a culturally rooted concept reflecting interpersonal trust and support, has been shown to buffer the negative impacts of workplace stressors (Yang et al. 2024). High‐quality guanxi may weaken the direct relationship between workplace harassment and turnover intention by providing emotional and professional support through strong supervisor–subordinate relationships.

Hypothesis 4 *The quality of supervisor–subordinate guanxi moderates the indirect relationship between workplace harassment and employees' turnover intention, mediated by organizational tolerance*..

This hypothesis extends Hypothesis 2 by incorporating the moderating effect of guanxi on the mediation pathway. Specifically, it is proposed that the mediating effect of organizational tolerance will vary depending on the quality of supervisor–subordinate guanxi. High‐quality guanxi is expected to mitigate the negative effects of perceived organizational tolerance on the relationship between harassment and turnover intention, consistent with cultural theories emphasizing interpersonal relationships in Chinese organizational contexts.

**FIGURE 1:**
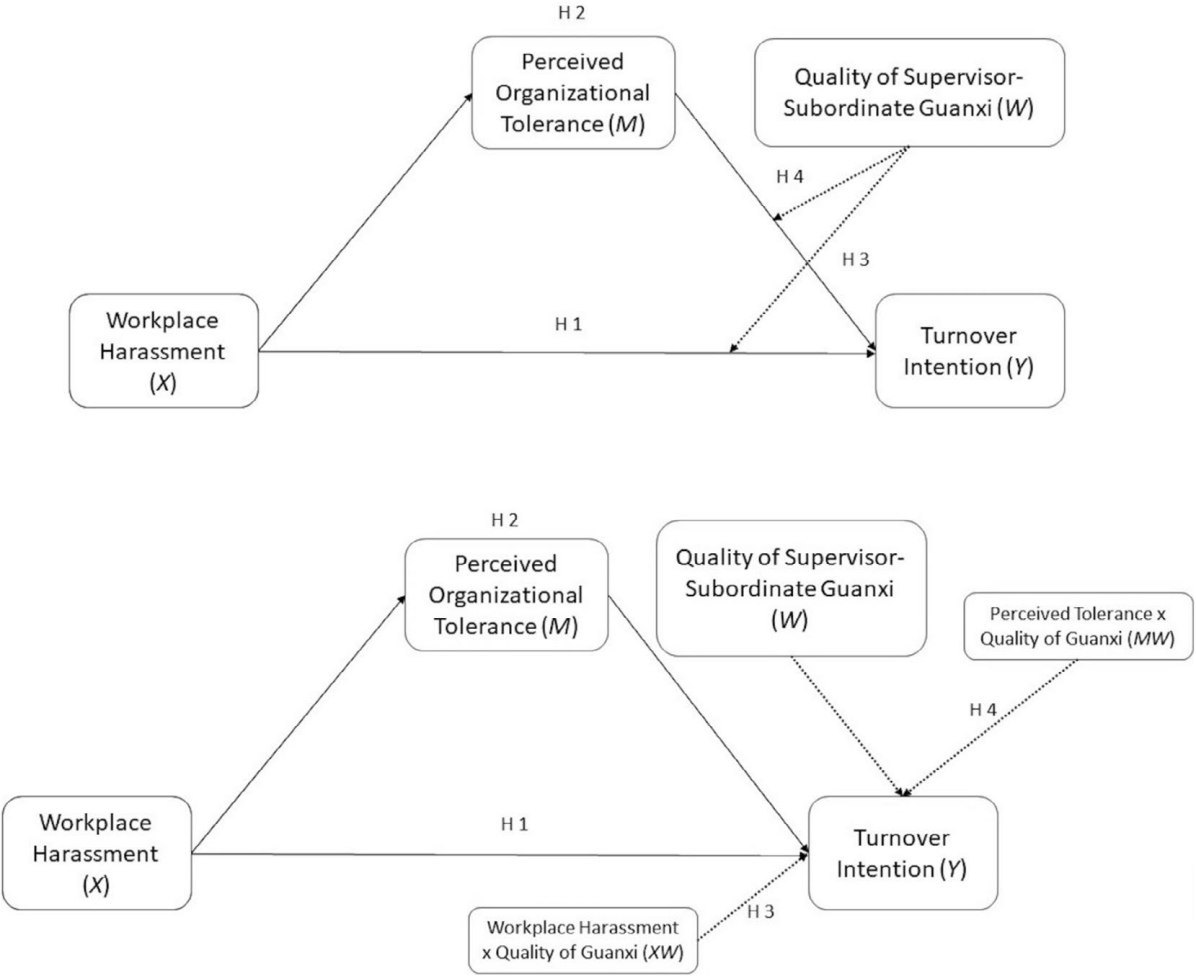
Research model. Conceptual and statistical diagram.

Figure 1 displays the research model with the hypotheses, including both the conceptual and the statistical diagrams.

## 2 Method

### 2.1 Participants

The sample for this study consisted of 821 law enforcement officers from various agencies across China. Among the participants, 480 were male (58.5%) and 341 were female (41.5%), reflecting the male predominance typically observed in this sector of law enforcement. The sample size was determined based on practical considerations, including the availability of participants and the scope of the study. While no formal power analysis was conducted prior to data collection, the sample size is sufficiently large to ensure robust statistical analyses and meaningful effect size estimation. Educationally, the majority of the officers have attained a high level of formal education, with 746 officers (90.9%) possessing degrees in criminal justice, law, public administration, or advanced degrees such as a Master's or MBA. The remaining officers have lower levels of formal education, with 44 (5.4%) having completed an associate's or bachelor's degree in criminal justice, law enforcement, public administration, or related fields and 31 (3.8%) having finished a high school diploma.

The officers are employed across various types of law enforcement agencies. A notable portion, 372 officers (45.3%), works in agencies that are private but include public capital or are part of a foundation, reflecting a mixed funding structure. Another 283 officers (34.5%) are part of public agencies, and 166 officers (20.2%) serve in entirely private law enforcement bodies.

Professionally, the data indicate a high proportion of officers in senior or managerial positions, with 523 individuals (63.7%), representing senior or managerial roles. This category includes high‐ranking positions such as captains, commanders, or administrative roles within the law enforcement agencies. Additionally, 277 officers (33.7%) hold mid‐level positions (officers who hold supervisory roles such as sergeants or lieutenants, or specialists with significant responsibility such as forensic analysts or senior detectives), and a smaller fraction are in lower ranks, with 13 officers (1.6%) who have moved beyond entry‐level but are not yet in positions of significant responsibility (patrol officers or junior detectives) and 8 officers (1.0%) in entry‐level positions (recently graduated from training academies).

Demographically, the officers are predominantly middle‐aged, with an average age of 52.51 years and a standard deviation of 4.97 years. The officers exhibit a considerable range in tenure at their respective agencies, with an average tenure of 26.36 years but a large variation, as indicated by a standard deviation of 28.05 years. This tenure spans from a minimum of 6 years to several decades, highlighting a broad spectrum of experience levels within the law enforcement community. The present sample presents a somewhat biased portion of the population as being a highly educated and experienced cohort of law enforcement officers in China, but serving across a diverse array of agencies and holding varying ranks within the law enforcement hierarchy.

### 2.2 Procedure

In the present study, the survey was administered through social networking platforms known for their professional use, specifically WeChat, Zhihu (知乎) and LinkedIn. The choice of these platforms was based on their popularity and widespread acceptance among professionals in China, which provided a suitable environment for engaging with law enforcement officers. The survey was designed to include questions that were clear, unbiased, and relevant to the professional experiences and opinions within the law enforcement community. These questions were also crafted to be culturally sensitive to the Chinese context, ensuring that respondents felt comfortable and understood.

The ethical standards upheld during the survey process were stringent, and ethical approval has been obtained from the Institutional Review Board of the East China University of Political Science and Law. The study was designed and conducted in accordance with the World Medical Association Declaration of Helsinki and local legislation.

Before participation, all respondents were provided with detailed information about the purpose of the survey, the use of the collected data, and the duration of the survey. To protect their privacy and professional identities, the confidentiality of responses was guaranteed, and participants were required to give informed consent by answering the first three questions of the survey.

To reach a diverse and representative sample, the survey was disseminated with the involvement of the East China University of Political Science and Law and other educational institutions that specialize in criminal justice and security management, allowing the survey to tap into a demographic that includes both seasoned practitioners and newcomers to the field.

Participants received an email with instructions and the link to fill out the survey through a recognized Chinese website for research, Wenjuanxing (Questionnaire Star). The survey only collects responses, but it does not store any additional information, such as participants' IPs or any personal or confidential data.

### 2.3 Instruments

#### 2.3.1 Workplace Harassment

The study employed the Chinese Workplace Bullying Scale (CWBS) developed by Li et al. (2012) to assess experiences of workplace harassment. This scale comprises 14 items encapsulating three distinct factors: personal attack, work pressure, and social isolation. Each item is rated on a 5‐point Likert scale ranging from 1 (*never*) to 5 (*continuously*). Sample items include “Someone withholds information deliberately to hinder my work performance,” “I am requested to work overtime,” and “I am overly teased or experience others' sarcasm.”

#### 2.3.2 Perceived Organizational Tolerance of Workplace Harassment

Tolerance toward Workplace harassment was measured using the Perceived Organizational Tolerance (POT) Scale (Perez‐Larrazabal et al. 2019). The original instrument includes 17 items that evaluate the intolerance of harassment myths and behaviors. The scale was adapted to focus on organizational tolerance, instead of intolerance, reversed the content of the items. Responses are gathered on a 5‐point Likert scale from 1 (*strongly disagree*) to 5 (*strongly agree*), where higher scores indicate a greater tolerance of harassment. Examples of items are: “My organization has clearly explained to us how to act if we suffer workplace harassment” (reversed). “If I report a harassment incident, my organization will not try to deal with it diligently.” “In my organization, the workers who have reported workplace harassment have suffered reprisals.” “My organization will try to stop me from reporting a workplace harassment incident.”

#### 2.3.3 Turnover Intention

Turnover intention was gauged using the Turnover Intention Scale (TIS‐6), a shortened version of the original fifteen‐item Turnover Intention Scale. This version includes items 1, 3, 4, 6, 7, and 8 from the original scale. Examples of items are “How often have you considered leaving your job?” and “How often do you look forward to another day at work?”

#### 2.3.4 Quality of Supervisor–Subordinate Guanxi

The quality of supervisor–subordinate guanxi was measured using a scale developed by Yang and Lau (2015). This scale consists of six items rated on a 5‐point Likert scale from 1 (*strongly disagree*) to 5 (*strongly agree*). Sample items include interactions such as “Your Chinese supervisor invites you for lunch/dinner” and personal exchanges like “Share with my supervisor about my thoughts and feelings.”

#### 2.3.5 Sociodemographic Variables

Gender was assessed as a categorical variable, coded nominally as 1 for males and 2 for females. The professional category was assessed as an ordinal variable, reflecting participants' levels of responsibility within their organization. Categories included: (1) recently graduated from training academies, (2) patrol officers or junior detectives, (3) officers in mid‐level positions, and (4) senior or managerial positions. These variables were included in the analysis as covariates to account for their potential influence on the relationships among workplace harassment, organizational tolerance, and turnover intention.

### 2.4 Data Analyses

First, descriptive statistics for all variables were computed to understand the central tendencies and dispersions using SPSS 29.01. version. This step provided an initial overview of the data and ensured that the variables were properly distributed and suitable for further analysis. Data cleaning involved examining missing values and outliers; no cases required exclusion as the data were complete and met the assumptions for the planned analyses. Following this, Pearson correlation analyses were performed to explore the bivariate relationships between the key variables: workplace harassment, organizational tolerance, quality of supervisor–subordinate guanxi, and turnover intention. These correlations helped identify significant associations and provided a preliminary understanding of the interconnections among the variables.

To test the hypotheses involving mediation and moderation effects, a moderated mediation analysis was conducted using Model 15 from the PROCESS macro for SPSS (Hayes 2013). This model was selected as it allows for examining both mediation and moderation simultaneously, offering a robust framework for testing the hypothesized relationships. Workplace harassment served as the predictor variable (*X*), organizational tolerance was the mediator (*M*), the quality of supervisor–subordinate guanxi was the moderator (*W*), and turnover intention was the outcome variable (*Y*). Gender (coded as a nominal variable) and professional category (coded as an ordinal variable) were included as covariates to account for their potential influence on the results. The analysis was performed in two stages. First, the direct and indirect effects of workplace harassment on turnover intention through organizational tolerance were assessed to test the mediation hypothesis. Second, the moderating role of the quality of supervisor–subordinate guanxi was examined by testing the interaction effects on both the direct path (workplace harassment to turnover intention) and the indirect path (through organizational tolerance). This comprehensive approach ensured that both direct and conditional indirect effects were thoroughly evaluated, providing insights into the complex dynamics between workplace harassment, organizational tolerance, quality of supervisor–subordinate guanxi, and turnover intention.

## 3 Results

### 3.1 Correlational Analyses

The correlational analyses were conducted to investigate the relationships between turnover intention (*Y*), workplace harassment (*X*), organizational tolerance (*M*), and the quality of supervisor–subordinate guanxi (*W*). The Pearson correlation coefficients, along with their significance levels, provide insights into these relationships.

First, the analysis revealed a significant positive correlation between turnover intention and workplace harassment (*r* = 0.375, *p* < 0.001). This finding suggests that as workplace harassment increases, employees are more likely to have higher turnover intentions. The strength of this correlation indicates a moderate relationship, emphasizing the impact of negative workplace experiences on employees' decisions to leave the organization.

Additionally, a significant positive correlation was found between turnover intention and organizational tolerance (*r* = 0.301, *p* < 0.001). This correlation implies that higher perceived tolerance of inappropriate behaviors within the organization is associated with increased turnover intention. Employees who perceive their organization as more tolerant of negative behaviors are more inclined to consider leaving their jobs.

The quality of supervisor–subordinate guanxi showed a different pattern. There is a significant negative correlation between turnover intention and the quality of supervisor–subordinate guanxi (*r* = −0.178, *p* < 0.001). This indicates that better quality relationships between supervisors and subordinates are linked to lower turnover intentions. Strong, positive relationships with supervisors can thus act as a protective factor against turnover.

Interestingly, the correlation between workplace harassment and organizational tolerance was also significant and positive (*r* = 0.200, *p* < 0.001), suggesting that higher levels of workplace harassment are perceived in environments with greater organizational tolerance of such behaviors. However, the quality of supervisor–subordinate guanxi was not significantly correlated with workplace harassment (*r* = −0.050, *p* = 0.152), indicating that the quality of these relationships does not necessarily impact the occurrence of harassment.

Finally, there was a significant positive correlation between organizational tolerance and the quality of supervisor–subordinate guanxi (*r* = 0.158, *p* < 0.001). This finding suggests that environments with higher organizational tolerance may also foster better‐quality supervisor–subordinate relationships, although this relationship is relatively weak.

In summary, the correlational analyses highlight important dynamics between workplace factors and turnover intention. Workplace harassment and organizational tolerance are positively associated with turnover intention, while the quality of supervisor–subordinate guanxi has a protective effect. These insights underscore the need for organizations to address workplace harassment and foster strong supervisor–subordinate relationships to reduce turnover intentions.

The reliability of the measurement instruments used in the study was evaluated using Cronbach's alpha, with values reported on the diagonal of Table 1. The Cronbach's alpha values indicate acceptable to excellent internal consistency for the scales employed. Specifically, the reliability for Turnover Intention (*α* = 0.79) and Workplace Harassment (*α* = 0.78) demonstrates solid internal consistency, reflecting the robustness of these measures in capturing their respective constructs. Organizational tolerance exhibited excellent reliability (*α* = 0.87), suggesting the items effectively measure perceptions of tolerance toward workplace harassment. Last, the Quality of Supervisor–Subordinate Guanxi scale displayed acceptable reliability (*α* = 0.76), indicating consistent measurement of relational dynamics. These results confirm that the instruments used in this study are reliable for examining the relationships between the variables under investigation.

**TABLE 1:** Descriptive statistics and Pearson correlations.

Variable	Mean	SD	1	2	3	4
1. Turnover intention	1.99	0.69	*0.79*			
2. Workplace harassment	3.31	0.68	0.375**	*0.78*		
3. Organizational tolerance	2.99	0.84	0.301**	0.200**	*0.87*	
4. Quality of supervisor–subordinate guanxi	2.46	0.50	−0.178**	−0.050	0.158**	*0.76*

*Note:* Cronbach's alphas values are in the diagonal in italics.

** *p* < 0.01 (2‐tailed).

### 3.2 Hypotheses Testing

In this section, we examine the results of our hypotheses testing to understand the direct and mediated relationships between workplace harassment, organizational tolerance, turnover intention, and the moderating role of supervisor–subordinate guanxi, including gender and professional category as covariates.

Hypothesis 1 *Workplace harassment directly and positively impacts employees' turnover intention*.

The results support Hypothesis 1. The analysis reveals a significant positive direct effect of workplace harassment on turnover intention (*β* = 0.3855, *p* < 0.001), even after accounting for the effects of gender and professional category. Gender (*β* = 0.3002, *p* < 0.001) and professional category (*β* = 0.1390, *p* = 0.002) were also significant predictors of turnover intention. These findings suggest that higher levels of workplace harassment are associated with an increase in employees' turnover intention and that demographic factors also contribute to turnover intention (Table 2).

**TABLE 2:** Model summary and coefficients for organizational tolerance.

Model summary	*R* ^2^	MSE	*F*	df1	df2	*p*
	0.3047	0.5025	50.9035	7	813	< 0.001
Predictor	*B*	SE	*t*	*p*	LLCI	ULCI
Constant	2.0937	0.1888	11.0874	< 0.001	1.7230	2.4643
Workplace harassment	0.3855	0.0398	9.6788	< 0.001	0.3073	0.4636
Gender	0.3002	0.0535	5.6094	< 0.001	0.1952	0.4053
Professional category	0.1390	0.0449	3.0938	0.002	0.0508	0.2271

Abbreviations: df.: degrees of freedom; MSE: mean squared error.

These results highlight the robust relationship between workplace harassment and turnover intention while also acknowledging the additional contributions of demographic factors.

Hypothesis 2 *Organizational tolerance mediates the relationship between workplace harassment and employees' turnover intention*.

Hypothesis 2 is supported by the data. The mediation analysis shows that workplace harassment has a significant positive effect on organizational tolerance (*β* = 0.2214, *p* < 0.001), and organizational tolerance, in turn, significantly affects turnover intention (*β* = 0.2372, *p* < 0.001). The indirect effect of workplace harassment on turnover intention through organizational tolerance is significant, confirming that organizational tolerance acts as a mediator in the relationship between workplace harassment and turnover intention. Additionally, gender (*β* = 0.3002, *p* < 0.001) and professional category (*β* = 0.1390, *p* = 0.002) were significant covariates in the model, highlighting the influence of demographic factors (Table 3).

**TABLE 3:** Model summary and coefficients for turnover intention.

Model summary	*R* ^2^	MSE	*F*	df1	df2	*p*
	0.3047	0.5025	50.9035	7	813	< 0.001
Predictor	*B*	SE	*t*	*p*	LLCI	ULCI
Constant	2.0937	0.1888	11.0874	< 0.001	1.7230	2.4643
Workplace harassment	0.3855	0.0398	9.6788	< 0.001	0.3073	0.4636
Organizational tolerance	0.2372	0.0394	6.0258	< 0.001	0.1599	0.3144
Quality of supervisor–subordinate guanxi	−0.4078	0.0510	−7.9965	< 0.001	−0.5078	−0.3077
Harassment × guanxi	−0.0312	0.0711	−0.4390	0.6608	−0.1709	0.1084
Tolerance × guanxi	−0.4561	0.0753	−6.0537	< 0.001	−0.6040	−0.3082
Gender	0.3002	0.0535	5.6094	< 0.001	0.1952	0.4053
Professional category	0.1390	0.0449	3.0938	0.002	0.0508	0.2271

Abbreviations: df.: degrees of freedom; MSE: mean squared error.

These results confirm that organizational tolerance mediates the relationship between workplace harassment and turnover intention while demonstrating the contributions of demographic factors such as gender and professional category.

Hypothesis 3 *Quality of supervisor*–*subordinate guanxi moderates the direct relationship between workplace harassment and employees' turnover intention*.

The results provide partial support for Hypothesis 3, as Table 4 shows. The interaction term between workplace harassment and the quality of supervisor–subordinate guanxi (workplace harassment × supervisor–subordinate guanxi) was not significant (*β* = −0.0312, *p* = 0.6608), suggesting that the quality of supervisor–subordinate guanxi does not significantly moderate the direct relationship between workplace harassment and turnover intention.

**TABLE 4:** Conditional direct effects of workplace harassment on turnover intention.

Guanxi level	Effect	SE	*t*	*p*	LLCI	ULCI
−0.5005	0.4011	0.0430	9.3180	< 0.001	0.3166	0.4856
0.0000	0.3855	0.0398	9.6788	< 0.001	0.3073	0.4636
0.5005	0.3698	0.0621	5.9564	< 0.001	0.2480	0.4917

**FIGURE 2:**
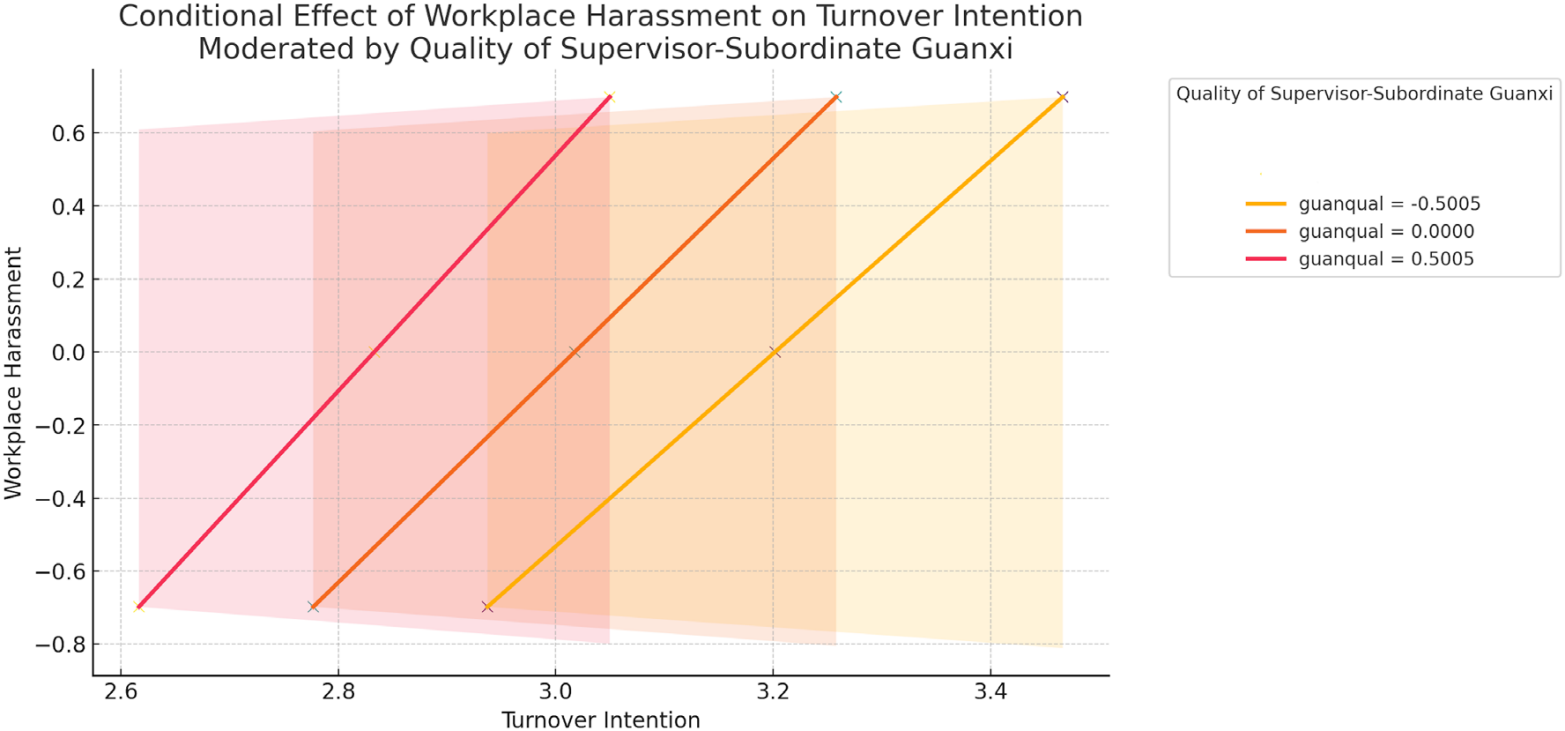
Conditional effects of workplace harassment on turnover intention moderated by guanxi quality.

However, the conditional direct effects indicate that the strength of the direct relationship varies with different levels of guanxi quality, as Table 4 shows. Specifically, the direct effect of workplace harassment on turnover intention decreases as the quality of supervisor–subordinate guanxi increases, but this moderation is not statistically significant. Additionally, gender (*β* = 0.3002, *p* < 0.001) and professional category (*β* = 0.1390, *p* = 0.002) were significant covariates in the model, highlighting the influence of demographic factors. Figure 2 illustrates the conditional effects of workplace harassment at different levels of guanxi quality.

Hypothesis 4 *The quality of supervisor–subordinate guanxi moderates the indirect relationship between workplace harassment and employees' turnover intention, mediated by organizational tolerance*.

The results support Hypothesis 4, as Table 5 shows. The moderated mediation analysis indicates that the quality of supervisor–subordinate guanxi moderates the indirect effect of workplace harassment on turnover intention through organizational tolerance. The index of moderated mediation is significant (index = −0.1010, BootSE = 0.0201, BootLLCI = −0.1422, BootULCI = −0.0627), indicating that the strength of the mediation effect changes at different levels of guanxi quality.

**TABLE 5:** Conditional indirect effects of workplace harassment on turnover intention via organizational tolerance.

Guanxi level	Effect	BootSE	Boot LLCI	Boot ULCI
−0.5005	0.1030	0.0178	0.0685	0.1383
0.0000	0.0525	0.0116	0.0315	0.0766
0.5005	0.0020	0.0124	−0.0220	0.0268

Moderated mediation	Index	BootSE	Boot LLCI	Boot ULCI
Guanxi	−0.1010	0.0201	−0.1422	−0.0627

**FIGURE 3:**
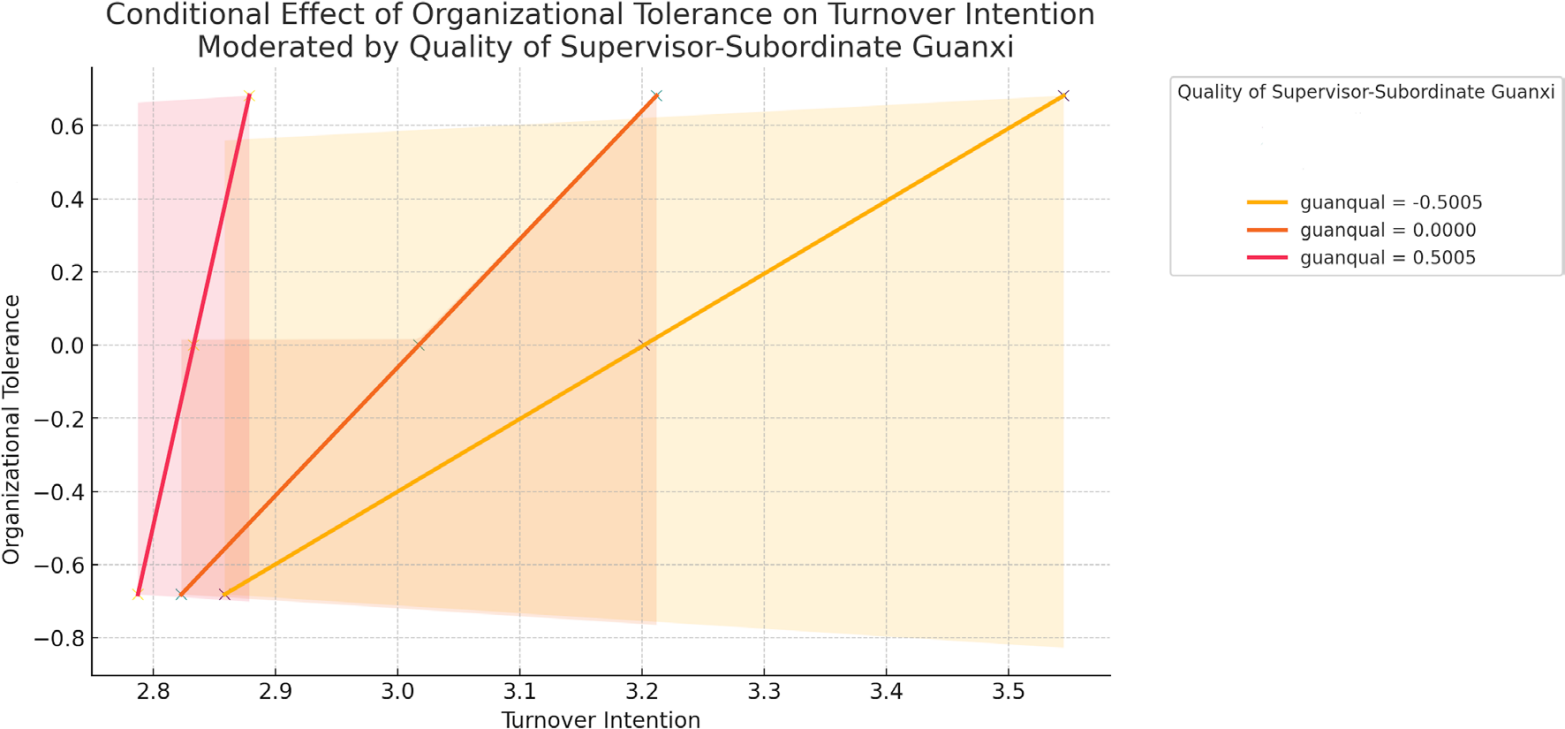
Conditional effect of organizational tolerance on turnover intention moderated by quality guanxi.

These results confirm that the quality of supervisor–subordinate guanxi significantly moderates the indirect relationship between workplace harassment and turnover intention. Specifically, as guanxi quality increases, the mediating effect of organizational tolerance diminishes. As Table 5 shows, the indirect effect is stronger when the quality of supervisor–subordinate guanxi is lower and diminishes as the guanxi quality improves. Figure 3 shows the conditional effects of organizational tolerance on turnover intention at the levels of guanxi quality.

In conclusion, the hypotheses testing provides comprehensive insights into the complex dynamics between workplace harassment, organizational tolerance, turnover intention, and the moderating role of supervisor–subordinate guanxi, even accounting for the influences of demographic factors as gender and professional category. These results confirm that the relationships among the primary variables hold even after accounting for demographic differences, emphasizing the generalizability and validity of the findings. These results highlight the importance of addressing workplace harassment and fostering strong supervisor–subordinate relationships to mitigate turnover intentions among employees.

## 4 Discussion

The findings from our study provide a nuanced understanding of the impact of workplace harassment on employees' turnover intentions, highlighting the mediating role of organizational tolerance and the moderating effect of supervisor–subordinate guanxi. Our results strongly support Hypothesis 1, demonstrating that workplace harassment has a significant positive direct effect on employees' turnover intentions. This finding aligns with previous research that underscores the detrimental effects of workplace harassment on employee retention in different countries (Ribeiro et al. 2024). For instance, Einarsen et al. (2020) found that employees subjected to harassment are more likely to consider leaving their jobs. This relationship is attributed to the psychological and emotional toll harassment takes on employees, leading to job dissatisfaction and a desire to exit the harmful environment.

The data also support Hypothesis 2, indicating that organizational tolerance mediates the relationship between workplace harassment and turnover intention. The mediation analysis revealed that workplace harassment positively affects organizational tolerance, and organizational tolerance, in turn, influences turnover intention. This suggests that in environments where harassment is tolerated, employees are more likely to leave. This finding is consistent with Houshmand et al. (2012), who highlighted that organizational climate plays a crucial role in either exacerbating or mitigating the effects of harassment on employee outcomes (Priesemuth and Schminke 2024). Moreover, workplace harassment is not an isolated phenomenon; it occurs within a group context. The working conditions, group‐level relationships, and reactions of group members to victimization provide a backdrop that can exacerbate or mitigate harassment activities (Rosander and Nielsen 2024). For instance, Opotow (2018) introduced the concept of moral frontiers, suggesting that group members perceived as highly integrated are more likely to be protected from harassment, whereas excluded members are more vulnerable. These dynamics further highlight the importance of organizational climate and group‐level interactions (Dvir and Nagar 2024).

Hypothesis 3 received partial support, revealing nuanced complexities in the moderating role of guanxi. Although the interaction term between workplace harassment and supervisor–subordinate guanxi was not statistically significant, indicating that guanxi does not significantly moderate the direct effect of harassment on turnover intention, the conditional effects suggest an interesting trend. Specifically, the strength of the direct relationship between workplace harassment and turnover intention decreases as guanxi quality improves, even if the moderation effect itself is not significant. This finding implies that strong supervisor–subordinate guanxi might provide a buffering effect, mitigating some of the adverse consequences of workplace harassment. However, the absence of a statistically significant moderation suggests that the protective role of guanxi in directly reducing turnover intention may be limited.

This nuanced result aligns with previous research (Wang 2019), which highlighted the conditional role of supervisor relationships in moderating workplace dynamics. Wang's work suggests that while high‐quality supervisor–subordinate relationships can offer emotional and professional support, they may not fully counteract the broader organizational and structural factors that drive turnover intention, such as perceived organizational tolerance of harassment or systemic power imbalances.

This highlights the complexity of interpersonal relationships in hierarchical and high‐stress environments like law enforcement. Unlike private sector organizations, where informal networks often hold significant sway, law enforcement agencies operate within rigid chains of command. Even strong interpersonal bonds may be insufficient to fully address or offset the negative impacts of workplace harassment, emphasizing the importance of organizational policies and structural changes in mitigating turnover intentions.

The observed trend raises further questions about nonlinear or context‐specific factors that could influence the relationship between workplace harassment, guanxi, and turnover intention. For instance, guanxi may operate differently at extreme levels of harassment or in environments with particularly high organizational tolerance. Future research could explore these nonlinear dynamics to gain a more comprehensive understanding of guanxi's role in workplace dynamics.

The study also provides strong support for Hypothesis 4, showing that the quality of supervisor–subordinate guanxi moderates the indirect effect of workplace harassment on turnover intention through organizational tolerance. The significant index of moderated mediation indicates that this mediation effect varies with guanxi quality. Specifically, the mediation effect is stronger when guanxi quality is lower and diminishes with higher guanxi quality. This finding aligns with the research of Huo et al. (2012), who found that high‐quality supervisor–subordinate relationships could mitigate negative organizational behaviors and their consequences.

Our findings corroborate with several studies but also present unique insights. The direct relationship between workplace harassment and turnover intention is well‐supported in the literature, aligning with studies such as Ribeiro et al. (2024). However, the partial moderation effect by supervisor–subordinate guanxi highlights a more complex interaction than previously discussed (Emerson et al. 2020). In this line, recent criticisms have linked guanxi to gender perspectives and suggested that the male‐centered standardized routine of guanxi could derive in a tension between trust and harassment (Tang 2020). Specifically, as some working environments, such as law enforcement, could be characterized by a gender imbalance, workplace harassment, mainly in the form of sexual harassment, would appear more as a consequence of power distance (Rosander et al. 2023). Conversely, our findings on organizational tolerance as a mediator enrich the existing discourse by adding a layer of understanding about how organizational climates can perpetuate turnover intentions if harassment is tolerated. This aligns with and extends the findings of Houshmand et al. (2012).

The inclusion of gender and professional category as covariates in this study provided additional insights into the dynamics of workplace harassment, organizational tolerance, and turnover intention. Both demographic factors significantly influenced the mediation and moderation effects examined in Hypothesis 4, highlighting their importance in understanding the broader context of workplace dynamics.

Gender emerged as a significant predictor, consistent with prior research suggesting that women are more likely to experience and be adversely affected by workplace harassment, particularly in male‐dominated fields such as law enforcement (Glomb et al. 1997; Lim and Cortina 2005; Zhou, Nguyen, et al. 2024). Female officers in the study reported higher sensitivity to organizational tolerance of harassment, which likely exacerbated their turnover intentions. This finding underscores the importance of addressing gendered power imbalances within hierarchical organizations and tailoring interventions to mitigate the unique challenges faced by women in these settings.

Professional category, which reflects hierarchical rank, also significantly influenced the relationships examined in Hypothesis 4. Employees in lower‐ranking positions, such as patrol officers or junior detectives, demonstrated greater susceptibility to the negative effects of workplace harassment and perceived organizational tolerance. This aligns with previous studies indicating that power imbalances in hierarchical organizations disproportionately impact lower‐ranking employees, who may feel less empowered to challenge or report harassment (Ajuwa et al. 2024; Willness et al. 2007). Conversely, higher‐ranking officers may have greater access to resources and support systems, buffering them from some of the adverse effects of harassment.

These findings suggest that gender and professional category play pivotal roles in shaping employees' experiences of harassment and their responses to organizational tolerance. While this study focuses on the moderating effect of supervisor–subordinate guanxi, the significant contributions of these covariates highlight the need for a more intersectional approach to understanding workplace harassment. Interventions aimed at reducing turnover intention must account for these demographic variables, ensuring that support systems and policies are equitable and address the unique vulnerabilities of different employee groups.

In conclusion, the significant influence of gender and professional category reinforces the complexity of workplace harassment dynamics. These factors interact with organizational and cultural variables, shaping employees' perceptions of harassment, organizational tolerance, and their ultimate decision to remain or leave. Future research should further explore the intersection of demographic factors, organizational structures, and cultural norms to develop more targeted and effective strategies for fostering supportive and inclusive workplace environments.

To sum up, our study confirms that workplace harassment significantly increases turnover intentions among employees, with organizational tolerance serving as a key mediating factor. Although the direct moderating effect of supervisor–subordinate guanxi is not significant, its influence on the mediated relationship suggests that improving these relationships can reduce the adverse effects of harassment indirectly. These insights emphasize the importance of addressing workplace harassment comprehensively and fostering supportive supervisor–subordinate relationships to mitigate turnover intentions.

While this study provides valuable insights into the relationships among workplace harassment, organizational tolerance, supervisor–subordinate guanxi, and turnover intention in the Chinese law enforcement context, several limitations should be noted. First, although two of the four scales used in this study were developed specifically for the Chinese cultural context—the Chinese Workplace Bullying Scale (CWBS) (Li et al. 2010) and the Supervisor–Subordinate Guanxi Scale (Yang and Lau 2015)—the remaining instruments were adapted from Western measurement tools and translated into Chinese. While these scales have been validated in cross‐cultural contexts, there is a possibility that certain nuances specific to the Chinese cultural and organizational environment may not have been fully captured. For example, Western conceptualizations of workplace harassment and turnover intention may not entirely align with the norms, values, and hierarchical structures inherent in Chinese workplaces.

Second, the use of adapted scales may introduce subtle biases, as cultural factors such as guanxi and hierarchical relationships are deeply embedded in Chinese organizations and may differ from the assumptions underlying Western‐developed tools. This limitation highlights the need for future research to develop or refine measurement instruments that are culturally tailored to ensure greater applicability and relevance to Chinese organizational settings.

Despite these limitations, the inclusion of culturally specific constructs, such as guanxi and the CWBS, provides a strong foundation for understanding how workplace dynamics operate in a Chinese context. Future studies could further address this limitation by combining adapted instruments with qualitative methods, such as interviews or focus groups, to capture the depth of cultural influences on workplace harassment and turnover intentions.

Another limitation pertains to the high levels of education and experience among the participants. The majority of the sample holds advanced degrees and has substantial tenure in their positions, which might not accurately reflect the broader population of law enforcement officers. This overrepresentation of highly educated and experienced individuals could skew the results, characteristics that could also impact turnover intentions (Megeirhi et al. 2020). Subsequent studies should consider including participants with a wider range of educational backgrounds and varying levels of professional experience to obtain a more comprehensive understanding of the issue. In this vein, some studies have suggested that maladjustment or inadequate organizational socialization could derive higher levels of workplace harassment perceptions among newcomers (Ma et al. 2024).

Additionally, the study is geographically and culturally specific to China, which may limit the applicability of the findings to law enforcement contexts in other countries (Murdoch et al. 2022). Cultural factors unique to China, such as the concept of guanxi, play a significant role in workplace dynamics and may not be as relevant or influential in other cultural settings (Hofstede 2023). Future research should explore these relationships in different geographical and cultural contexts to assess the universality of the findings and to understand how cultural variations impact workplace harassment and turnover intentions (Blumenthal et al. 2023; Bowen and Blackmon 2003; Chen et al. 2023; Copenheaver et al. 2023; Crusto et al. 2024). Moreover, some additional variables could be included in future studies, as the public service motivation (Erten and Çögenli 2024) that can explain the lower levels of turnover intention among public employees, even under stressful working conditions (Wang et al. 2021; Xu et al. 2023).

## 5 Conclusion

This study provides a comprehensive examination of the relationships between workplace harassment, organizational tolerance, turnover intention, and the moderating role of supervisor–subordinate guanxi among law enforcement officers in China. The findings confirm that workplace harassment significantly increases employees' turnover intentions, highlighting the critical impact of harassment on employee retention, as recent reviews suggest (Liang 2024).

The study also reveals that organizational tolerance mediates the relationship between workplace harassment and turnover intention. In environments where harassment is tolerated, employees are more likely to consider leaving their jobs. This underscores the importance of organizational policies and cultures that do not condone harassment, emphasizing the need for stringent anti‐harassment measures and supportive reporting mechanisms.

Moreover, while the direct moderating effect of supervisor–subordinate guanxi on the relationship between workplace harassment and turnover intention was not statistically significant, the quality of this relationship does affect the mediated relationship. Specifically, high‐quality supervisor–subordinate relationships can reduce the negative impact of workplace harassment by influencing organizational tolerance and subsequently turnover intentions.

Overall, this study underscores the complex dynamics between workplace harassment, organizational tolerance, and turnover intentions, and the nuanced role of supervisor–subordinate guanxi. It highlights the necessity for organizations to address workplace harassment proactively and foster strong, supportive relationships between supervisors and subordinates to mitigate the adverse effects of harassment on employee turnover (Ford and Ivancic 2020; Kaur 2023; Perez‐Larrazabal et al. 2019). Future research should aim to explore these relationships in more diverse contexts and over longer periods to further understand and address the issues identified.

## Ethics Statement

The ethical standards upheld during the survey process were stringent, and Ethical approval has been obtained by the Institutional Review Board of the East China University of Political Science and Law. The study was designed and conducted in accordance with the World Medical Association Declaration of Helsinki and the local legislation.

## Consent

Before participation, all respondents were provided with detailed information about the purpose of the survey, the use of the collected data, and the duration of the survey. To protect their privacy and professional identities, the confidentiality of responses was guaranteed, and participants were required to give informed consent by answering the first three questions of the survey.

## Conflicts of Interest

The author declares no conflicts of interest.

## Data Availability

The data that support the findings of this study are available from the East China University of Political Science and Law, but restrictions apply to the availability of these data, which were used under license for the current study and so are not publicly available. The datasets used and/or analyzed during the current study are available from the corresponding author on reasonable request.
